# Immuno-Modulator Metallo-Peptide Reduces Inflammatory State in Obese Zucker Fa/Fa Rats

**Published:** 2014-09

**Authors:** Antonieta Gómez-Solís, Jorge Reyes-Esparza, Francisco García-Vázquez, Elizabeth Álvarez-Ayala, Lourdes Rodríguez-Fragoso

**Affiliations:** 1 Facultad de Farmacia, Universidad Autónoma del Estado de Morelos, Cuernavaca 62210, Morelos, México;; 2 Instituto Nacional de Pediatría, México city 04530, México

**Keywords:** Metabolic syndrome, Obesity, Inflammation, Peptide, Immunomodulator

## Abstract

Metabolic syndrome is a prothrombotic and proinflammatory chronic state. In obesity, the adipose tissue secretes various adipokines that take part in a variety of physiological and pathophysiological processes, including immunity and inflammation. Previous studies using a liver damage model treated with the immune-modulator metallo-peptide (IMMP) showed lessening in the degree of inflammation. Therefore, this study was set up to evaluate the anti-inflammatory effect of IMMP in obese Zucker fa/fa rats. We used Zucker-Lepr fa/fa and Zucker-Lean in this protocol. The groups received IMMP 50 ng/kg by i.p., three times per week for 8 weeks. Blood samples were collected by cardiac puncture and the serum was preserved at -80°C until analysis; the liver was excised and preserved in formaldehyde 4%. Analyses were performed to determine cytokine, insulin, glucose, triglyceride and cholesterol levels in serum, and histological analysis was also performed. IMMP treatment of obese rats resulted in decreased levels of proinflammatory cytokines (leptin, lL-6, IL-1betha, INF-gamma) and a chemokine (MCP-1), and increased levels of anti-inflammatory adipokine (adiponectin). In addition, treatment decreased the damage and hepatic steatosis generated in the tissue of obese rats. The IMMP exerted an anti-inflammatory effect in obese rats and therefore may be an effective and safe therapeutic alternative in the treatment of metabolic syndrome.

## INTRODUCTION

Metabolic syndrome is defined as a prothrombotic, proinflammatory chronic state, associated with endothelial dysfunction and accelerated atherogenesis. It is a cluster of multifactor risks that include atherogenic dyslipidemia, rises in blood pressure, rises of glucose in serum or the development of insulin resistance, and abdominal obesity (1). Metabolic syndrome affects more than a third of the U.S. and Mexican populations, predisposing them to the development of prediabetes or type 2 diabetes, and/or cardiovascular disease. The prevalence in adults from different populations varies between 20 to 40% and increases with age (2).

The inflammatory state in metabolic syndrome is quite peculiar, as it is not accompanied by an infection or signs of autoimmunity and no massive tissue damage seems to have occurred (3, 4). The extent of inflammatory activation is not large, so this chronic inflammation is classified as "low grade". Adipose tissue secretes various adipokines that take part, via endocrine, paracrine, autocrine and juxtacrine signaling in a wide variety of physiological and pathophysiological processes, including food intake, insulin sensitivity, sclerotic vascular processes, immunity and inflammation (5, 6).

Most of the products secreted by adipose tissue play a leading role in inflammation and immune responses. Namely, fat cells or adipocytes produce interleukin-6 (IL-6), tumor necrosis factor (TNF-α) and many other cytokines and hormones including leptin and adiponectin (7, 8). In addition, the increase of adipose tissue among the obese increases the production of inflammatory proteins, developing a proinflammatory state in the subject. Several studies have shown high levels of proinflammatory cytokines in the serum of obese patients, and this increase is related to the degree of obesity (9, 10).

Inmunomodulators are substances that can influence various components of the immune system. The thymic octapeptide Leu-Glu-Asp-Gly-Pro-Lys- Phe-Leu (L1EDGPKF L8) has been implicated in augmenting T-cell functions, such as the response to T-cell lectins and mixed lymphocyte reactions; it also increases interleukin-2 production by T-cells (11-14). The immune-modulator metallo-peptide (IMMP) is a molecular complex made of the synthetic octapeptide sequence leu-glu-asp-gly-pro-lys-leu-phe (L1EDGPKFL8) and zinc. Previous studies have shown that IMMP induces cell proliferation in rat thymocytes, human lymphocytes, Jurkat T cells and monocytic cells; it also induces phagocytosis in monocytic cells (THP-1 cells) (15). Previous studies in two models of liver damage have shown that IMMP is able to partially increase populations of T cells and decrease inflammation degree by decreasing production of IL-6 (16). This study sought to evaluated the anti-inflammatory effect of IMMP in obese Zucker fa/fa rats.

## METHODS

### Reagents

IMMP was synthesized by New England Peptide, Gardner, MA, USA. The commercial kits used in this experimental protocol were Glucose PAP, Triglycerides SDS and Cholesterol PAP (ELITech Group, Paris, France); Leptin, adiponectin and MCP-1 (Millipore, Missouri USA); Insulin (Crystal Chemic, USA); primary antibody and recombinant protein of IL-6, IL-10, IL4, TNF-α, IFN-γ and IL-1β (Peprotech Inc, NJ, USA); secondary antibody Anti-Rabbit IgG Peroxidase conjugated Affine Pure Goat (JacksonInmunoResearch, USA), and substrate 2,2´-Azino-bis(3-ethylbenzothiazoline 6-sulfonic acid) ABTS (Sigma-Aldrich, USA).

### Animals

Ten male Zucker-Lepr fa/fa rats and ten Zucker-Lepr Lean, weighing 120 g-150 g were purchased from Harlan Mexico, S.A. de C.V. Rats were randomly housed in four groups of 5 per cage under controlled conditions (24°C, 58% humidity and 12 h day/night cycle). Rats were fed with a Rodent Laboratory Chow diet and had access to food and water ad libitum. All procedures were approved by the Institutional Animal Care and Use Committee of the Veterinary Medical School at the National Autonomous University of Mexico. Experiments were conducted following the rules and principles set in the Guide for the Care and Use of Laboratory Animals (17).

### Pharmacological treatments and sample collection

Rats were randomly distributed into the following groups: 1) Lean: Zucker-Lepr Lean rats received 0.5 mL of PBS by i.p., three times per week for 8 weeks; 2) Lean+IMMP: Zucker-Lepr Lean rats received IMMP 50 ng/kg three times per week by i.p., three times per week for 8 weeks; 3) Obese: Zucker-Lepr fa/fa rats received 0.5 mL of PBS by i.p., three times per week for 8 weeks; and 4) Obese + IMMP: Zucker-Lepr fa/fa rats received IMMP 50 ng/kg by i.p., three times per week for 8 weeks. Groups of five rats each were euthanized with ether at the end of treatment. Blood samples were collected by cardiac puncture and centrifuged for 15 min at 3000 rpm. The serum was preserved at -80°C until analysis. The liver was excised and preserved in formaldehyde 4%.

### Determination of cytokine in serum

IL-6, TNF-α, IFN-γ, IL-1β, IL-10 and IL4 levels were analyzed by ELISA. Briefly, wells were coated with 100 μL of sample or standard and incubated overnight at 4°C. Wells were aspirated and washed and blocked with 150 μL of casein 5% for 60 min at room temperature. After washing, 100 μL of primary antibody (dilution 1:1000) were added to each well and incubated for 60 (IFN-γ, IL-6, and IL4 antibodies) or 120 min (TNF-α and IL-10 antibodies) at room temperature, followed by washing and an added 100 uL of secondary antibody (dilution 1:1500), incubated for 60 min at room temperature. After washing, 100 μL of substrate were added to each well for detection and incubated for 30 or 60 min at room temperature. Samples were quantified in a Multimode Plate Reader Victor X3, Perkin Elmer (USA) at 405 nm.

### Determination of insulin, leptin, adiponectin and MCP-1 in serum

Insulin, leptin, adiponectin and MCP-1 in serum were determined using a commercial kit in accordance with the manufacturer’s instructions (Crystal Chemic -USA-, for insulin; and Millipore, -USA- for leptin, adiponectin and MCP-1). The absorbance was quantified in a Multimode Plate Reader Victor X3, Perkin Elmer (USA).

### Determination of glucose, triglycerides and cholesterol in serum

Clinical chemical parameters (glucose, triglycerides and cholesterol) were determined in serum using a commercial kit in accordance with the manufacturer’s instructions (ELITech Group, Paris, France). The absorbance was quantified in a Multimode Plate Reader Victor X3, Perkin Elmer (USA).

### Histopathological analysis of liver

The liver tissue fragments were fixed in 4% formaldehyde solution, dissolved in phosphate-buffered saline (pH 7.4), dehydrated in alcohol and embedded in paraffin. Two-micrometer paraffin sections were stained with Hematoxylin and Eosin (H&E), and then subjected to histopathological examination. Images from each liver sample were digitalized using an Olympus IX2-UCB camera (Tokyo, Japan) at × 40.

### Statistical analysis

Data are expressed as means ± standard deviation. Significant differences were detected by one-way analysis of variance using SPSS 15.0 software. The Tukey test was applied and differences were considered significant at *p*<0.05.

## RESULTS

### IMMP modification of adipokine levels in Zucker fa/fa rats

**Figure 1:**
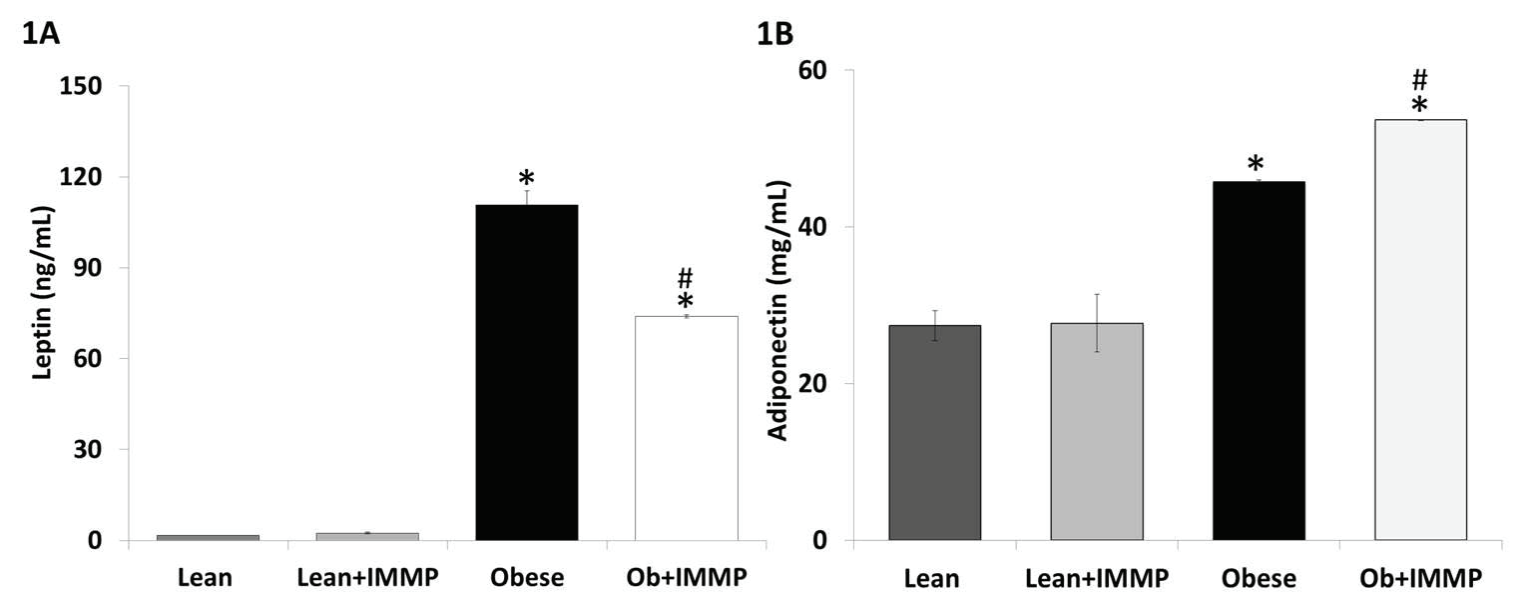
Effect of IMMP on adipokine levels in the serum of lean and obese rats treated during 8 weeks. A, Leptin; B, Adiponectin. The results are presented as mean ± S.D. from 5 animals in each group. ^*^ *p*<0.05 compared with Lean group; ^#^ *p*<0.05 compared with Obese group.

We started our evaluation of the IMMP effect by measuring its impact on two adipokines that are involved in the development of the obesity-related inflammation process. Leptin is regarded as a proinflammatory cytokine: its production is increased as the degree of obesity increases and adiponectin, an anti-inflammatory cytokine, is inhibited when levels of proinflammatory cytokines increase. Figure 1A shows the effect of IMMP on leptin levels: obese rats showed a 68-fold increased (*p*<0.05) when compared with lean rats. When obese rats were treated with IMMP for 8 weeks we observed a decrease (33%) in leptin levels when compared with untreated obese rats (*p*<0.05). On the other hand, we also saw an increase in the adiponectin levels of obese rats (1.7-fold) when compared to lean rats (*p*<0.05). When the obese rats were treated with IMMP, adiponectin levels were increased by 17% when compared with untreated obese rats (*p*<0.05) (Figure 1B). Lean rats treated with IMMP did not show changes in either adipokine.

### IMMP decreases proinflammatory cytokines in Zucker fa/fa rats

**Figure 2:**
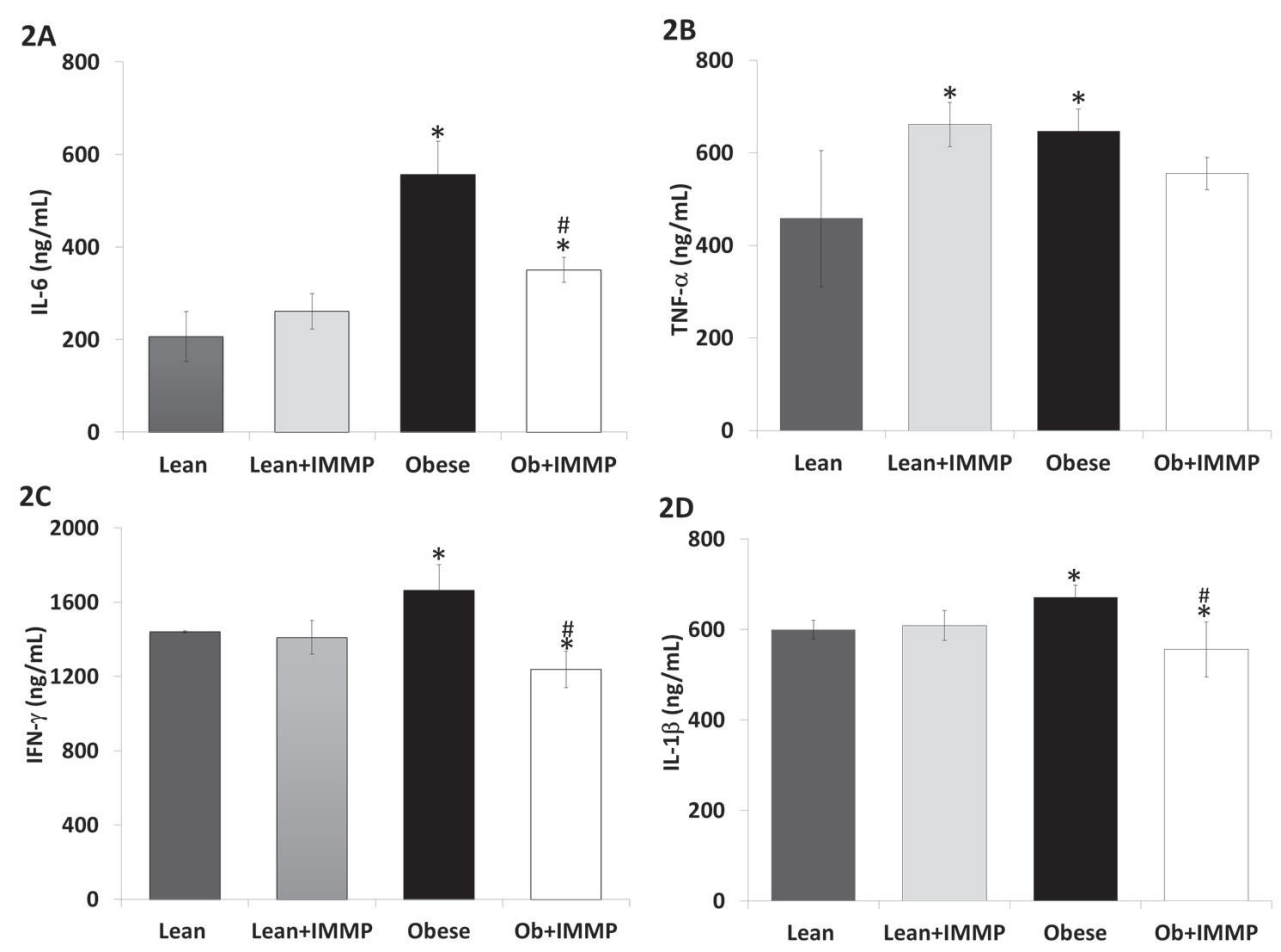
Effect of IMMP on proinflammatory cytokine levels in the serum of lean and obese rats treated during 8 weeks. A, IL-6; B, TNF-α; C, INF-γ and D, IL-1β. The results are presented as mean ± S.D. from 5 animals in each group. ^*^ *p*<0.05 compared with lean group; ^#^ *p*<0.05 compared with obese group.

To evaluate the anti-inflammatory effect of IMMP, we evaluated different pro-inflammatory cytokines, including IL-6, TNF-α, IFN-γ and IL-1β (Figure 2). When we analyzed IL-6 levels in obese rats we found 1.6-fold increase when compared to the lean rats (*p*<0.05). Obese rats treated with IMMP showed a decrease (37%) in IL-6 levels when compared to untreated obese rats (*p*<0.05). Levels of this cytokine showed no changes in lean rats treated with IMMP (Figure 2A). Figure 2B shows that TNF-α levels also increased (41%) in the obese group when compared to the lean rats (*p*<0.05). Obese rats treated with IMMP did not show modified cytokine levels. However, lean rats treated with IMMP increased TNF-α levels by 44% when compared to untreated lean rats (*p*<0.05).

Figure 2C shows IFN-γ levels in obese rats. We observed an increase (15%) of this cytokine in comparison to lean rats (*p*<0.05). Obese rats treated with IMMP showed a decrease (25%) in this cytokine’s levels when compared with untreated obese rats (*p*<0.05). Figure 2D shows IL-1β levels in obese rats. We can see there was a 12% increase when compared with lean rats (*p*<0.05). However, obese rats treated with IMMP showed a decrease in IL-1β levels (9%) when compared with untreated obese rats (*p*<0.05). Lean rats treated with IMMP did not show changes in levels of this cytokine.

### MCP-1 levels decreased in Zucker fa/fa rats treated with IMMP

**Figure 3:**
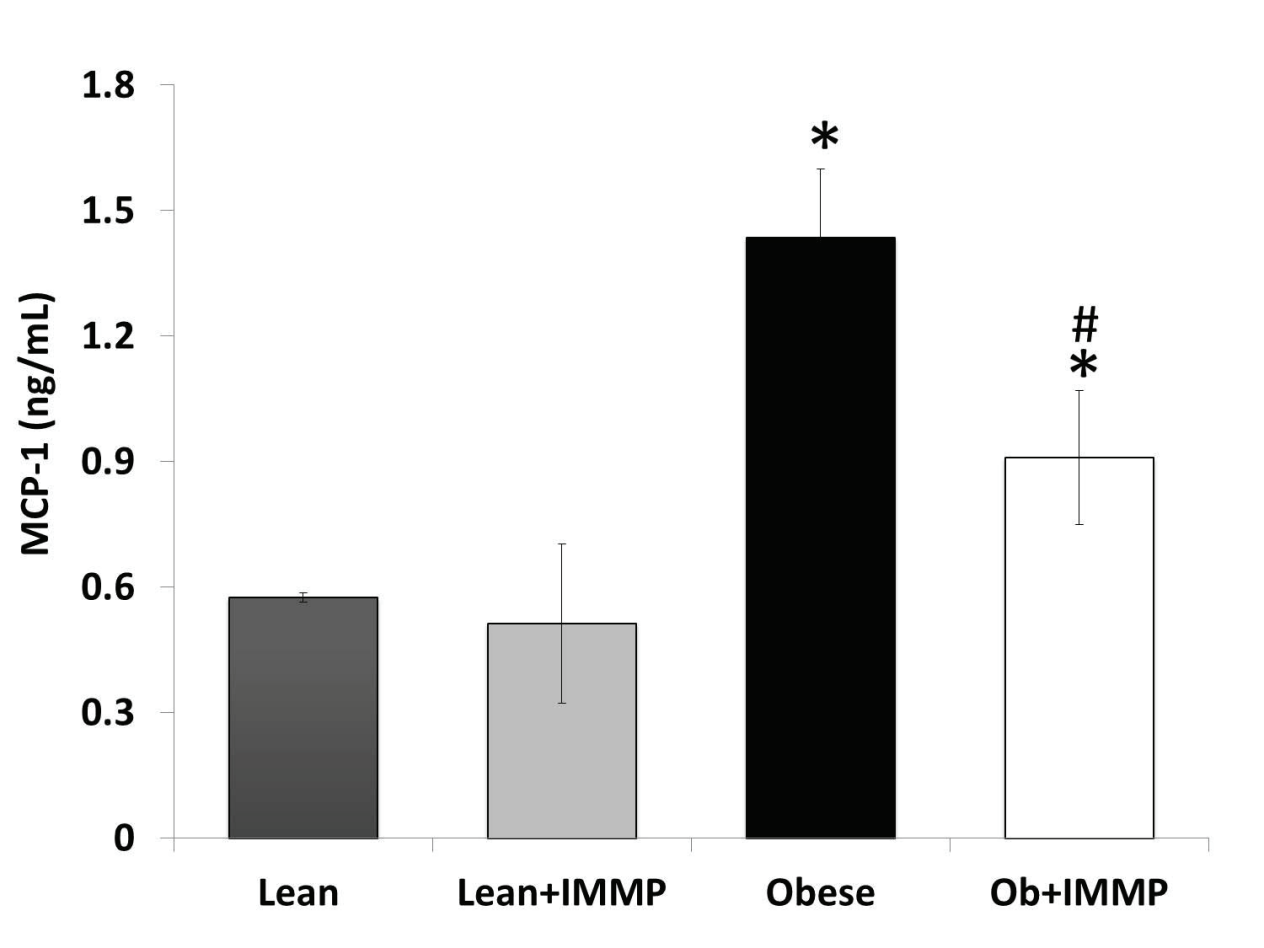
Effect of IMMP on MCP-1 levels in the serum of lean and obese rats treated during 8 weeks. The results are presented as mean ± S.D. from 5 animals in each group. ^*^ *p*<0.05 compared with lean group; ^#^ *p*<0.05 compared with obese group.

MCP-1 is a monocyte chemo-attractant protein. An increase in chemokine levels during the development of obesity has been observed, since its function is to attract macrophages in the adipose tissue and thereby increase inflammation across several tissues. Quantitation results of this chemokine are shown in Figure 3. We found a 2.5-fold increase in MCP-1 levels in obese rats when compared to the lean group (*p*<0.05). When the obese rats were treated with IMMP they showed a decrease (33%) in MCP-1 levels in comparison with untreated obese rats (*p*<0.05). No changes in MCP-1 levels were observed in lean rats treated with IMMP.

### IMMP did not modify anti-inflammatory cytokine levels in Zucker fa/fa rats

**Figure 4:**
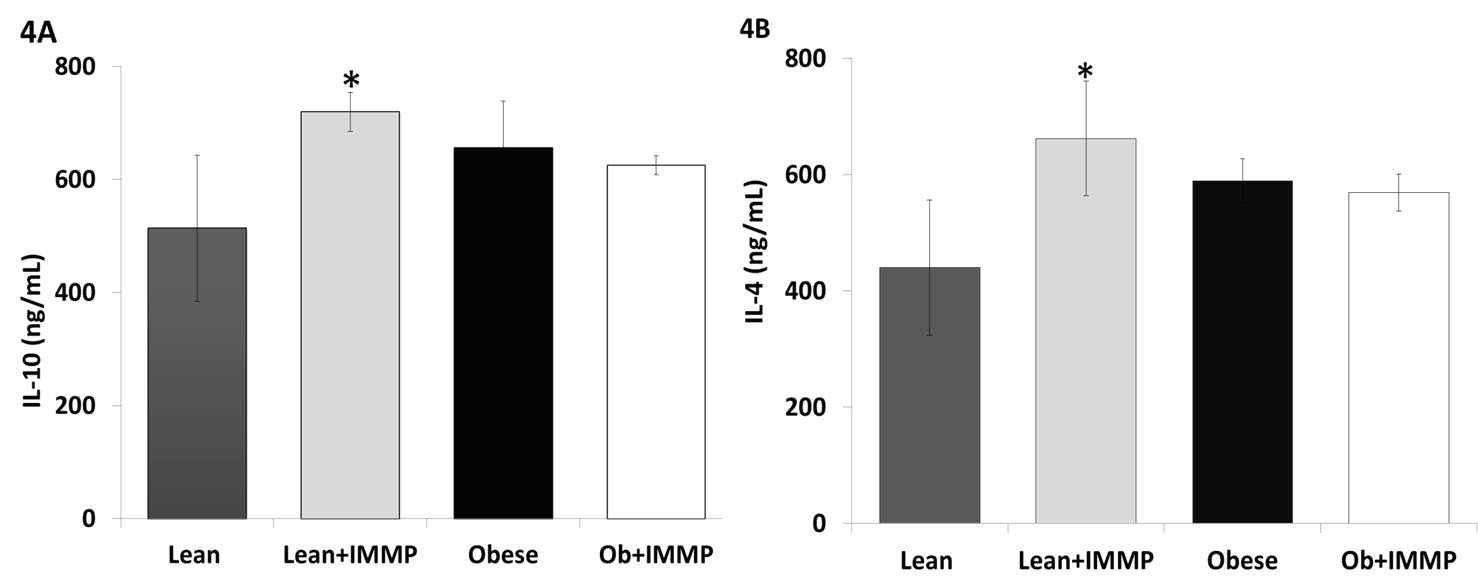
Effect of IMMP on anti-inflammatory cytokine levels in the serum of lean and obese rats treated during 8 weeks. A, IL-10; B, IL-4. The results are presented as mean ± S.D. from 5 animals in each group. ^*^ *p*<0.05 compared with lean group; ^#^ *p*<0.05 compared with obese group.

We decided to evaluate the effect of IMMP on anti-inflammatory cytokines (IL-10 and IL-4) in order to know if IMMP produces an anti-inflammatory effect in obese animals treated for 8 weeks. As we can see in Figure 4A, IL-10 levels did not show significant changes either in obese rats or obese rats treated with IMMP for 8 weeks. However, lean rats treated with IMMP showed an increase in IL-10 levels (26%) when compared with lean group (*p*<0.05). A similar pattern was observed regarding IL-4 levels across all groups (Figure 4B). Lean rats treated with IMMP showed an increase (50%) in the amount of this cytokine (*p*<0.05).

### IMMP did not modify clinical chemical parameters in Zucker fa/fa rats

Weight gain has been widely reported in the type of obese rats used in this work. We found an important increase in body-weight gain (282.47 ± 13.53 g) in obese rats after 8 weeks when compared to lean rats (158.66 ± 6.2) (*p*<0.05). Obese rats treated with IMMP did not show significant changes in in body-weight gain when compared with obese rats. A similar effect was observed in lean rats treated with IMMP (Table 1).

**Table 1:** Effect of IMMP on weight gain and serum levels of glucose, insulin, triglycerides and cholesterol after 8 weeks of evaluation

PARAMETERS	LEAN	LEAN+IMMP	OBESE	OB+IMMP

Weight Gain (g)	158.66 ± 6.27	176.10 ±15.99	282.47 ± 13.53^a^	296.25 ± 13.80^a^
Glucose (mg/dL)	82.21 ± 1.34	112.62 ± 6.98^a^	141.38 ± 16.58^a^	137.86 ± 18.38^a^
Insulin (ng/mL)	0.12 ± 0.02	0.20 ± 0.03	1.65 ± 0.04^a^	1.43 ± 0.33^a^
Triglycerides (mg/dL)	61.71 ± 5.43	66.25 ± 2.93	622.10 ± 16.89^a^	864.25 ± 192.06^a^ ^b^
Cholesterol (mg/dL)	46.11 ± 3.98	54.48 ± 1.59	83.59 ± 10.81^a^	77.70 ± 10.71^a^

Values are presented as mean ± S.D. from 5 animals in each group.

a *p*<0.05 compared with lean group;

b *p*<0.05 compared with obese group.

Analyzing the clinical chemical parameters we observed that obese rats had a 72% increase in glucose levels when compared to lean rats (*p*<0.05). No changes were observed in obese rats treated with IMMP. However, we found an increase of 36% in the glucose levels of lean rats treated with IMMP. The insulin serum levels increased significantly (14-fold) among obese rats when compared to lean rats, while obese rats treated with IMMP did not show significant changes in insulin levels when compared to obese rats. No changes in insulin serum level were observed in treated lean rats (Table 1). On the other hand, obese rats showed an important increase (10-fold) in serum triglycerides and (1.8-fold) in serum cholesterol when compared with lean rats. Obese rats treated with IMMP did not show a significant decrease in serum triglycerides and cholesterol when compared to untreated obese rats. Lean rats showed no changes in their parameters after treatment with IMMP (Table 1).

### Reduction of liver damage in IMMP treated Zucker fa/fa rats

**Figure 5:**
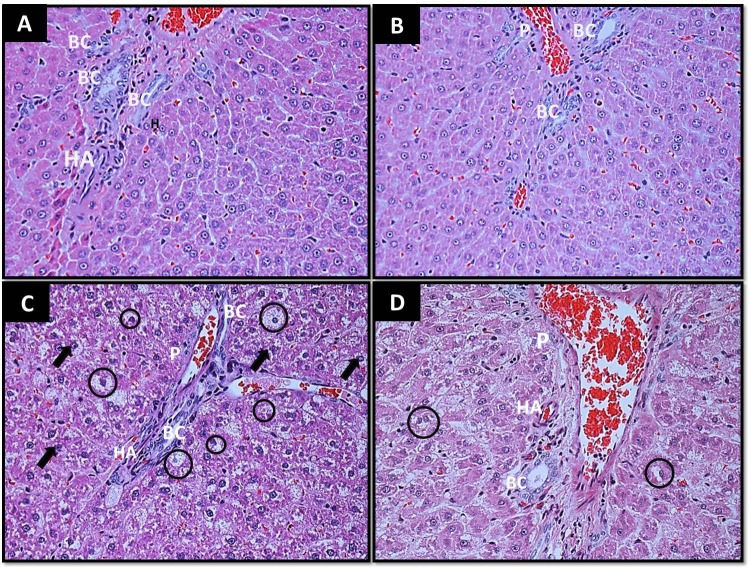
Effect of IMMP on the morphology of the liver. Liver slice A, Lean; B, Lean+IMMP; C, obese and D, obese+IMMP after 8 weeks of evaluation. Hematoxylin and Eosin staining, magnification × 40. P, Portal; HA, Hepatic artery; BC, bile duct; Circle, lipid vacuoles; Arrows, polymorphic nuclei.

Figure 5 shows representative histological changes in the liver of the different groups. By week 8, obese rats showed hepatocellular injury characterized by centrolobular, microvesicular fatty infiltration, ballooning degeneration and pleomorphic nuclei (Figure 5C). IMMP treatment during an 8 week period decreased liver damage in obese rats, reducing inflammation and fat infiltration as seen in Figure 5D. IMMP treatment in lean rats did not modify or alter the normal liver structure (Figure 5B).

## DISCUSSION

The clinical management of patients with metabolic syndrome is based on lifestyle changes to induce weight-loss, an increase in physical activity, and the implementation of a balanced diet. When this therapy is not effective, pharmacological therapy is employed. Due to the complexity of the treatment, its reduced adherence and the presence of various adverse effects, we must look for new treatment options for this condition. Here we propose the use of IMMP as an alternative treatment for this syndrome in a Zucker fa/fa obese rat model.

Metabolic syndrome model in Zucker fa/fa rats develops because of a mutation in the leptin receptor, which results in a deficiency in its signaling. These animals are characterized by obesity, hyperlipidemia, hyperphagia and hiperinsulinemia (18). Our study found that Zucker fa/fa rats became obese and gained twice the weight amount of Zucker lean rats. This weight gain has also been observed in different obesity models among which we can mention those induced by diets that are high in fats or carbohydrates, as well as genetic models such as ob/ob mice (19). The use of peptides or their agonists have been reported to exert an effect on food intake, thereby reducing body weight in animal models as well as clinical studies (20). Unlike these, IMMP did not decrease food intake in animals (data not shown), and there was therefore no decrease in weight gain. This could be due to the animal model employed, since the genetic modification of the leptin receptor alters the animal’s satiety point, making it difficult for the peptide to modify food intake.

The development of inflammation in obesity, both in experimental models and humans, involves the recruitment of macrophages and the production and release of inflammatory factors known as adipocytokines in the adipose tissue (8). These findings have revealed this organ does not only store energy but is also an endocrine organ (21). The most important adipocytokines modified during development of obesity are leptin and adiponectin. Leptin secretion is directly related to the individual’s amount of adipose tissue. Levels of this adipocytokine in the serum of non-obese subjects lies in a range of 1 to 15 ng/mL, and the range in subjects with a BMI ≥ 30 index may increase up to 30ng/mL (22, 23). Leptin levels in the obese rats employed in our study also increased significantly. These findings are consistent with other studies that employed this model, as well as others induced via hypercaloric diets. However, in these models, obesity is not as prominent as with Zucker rats (24). On the contrary, the group of obese rats that received the IMMP showed significantly reduced levels of this adipocytokine. This peptide-mediated effect has been demonstrated in a study of obese rats with caloric restriction (25). Leptin, in addition to playing a role in the regulation of weight and appetite, has a proinflammatory effect that increases the production of proinflammatory cytokines; for this reason, our treatment might have an anti-inflammatory effect (26).

Another important cytokine in obesity-related inflammation is adiponectin. In non-obese subjects, adiponectin levels range from 5 to 10 g/mL, while in obese subjects or sufferers of diabetes mellitus, these levels are inversely proportional to the BMI and the accumulation of visceral fat (27). However, adiponectin levels have been found high in studies involving Zucker fa/fa rats (28). In the model of diabetic Zucker rats, adiponectin levels diminished significantly (29). Conversely, in patients with osteoarthritis, the adiponectin levels in plasma were almost 100 times higher than in synovial fluid, and these levels showed an inverse correlation (30). The controversy regarding these behaviors is due to the degree of damage sustained by the animal. Among Zucker fa/fa rats, adiponectin attempts to counter the inflammatory process and therefore increases in obese rats. As for diabetic Zucker rats, the inflammatory process is ongoing and thus inhibits the secretion of anti-inflammatory cytokines, including adiponectin (31, 32). Treatment with IMMP increased adiponectin levels in obese rats without modifying levels of this cytokine in lean rats.

The inflammatory process in obesity also involves other important cytokines, including classic ones such as TNF-α and IL-6, chemokines such as MCP-1 and MIP-1α, and proteins involved in vascular homeostasis such as the inhibitor plasminogen 1 (PAI-1). In addition to participating in the inflammatory process, these cytokines also promote the development of insulin resistance and hypertriglyceridemia. Elevated levels of the inflammatory marker high-sensitivity C-reactive protein (hs-CRP) are associated with increased risk for CVD and diabetes mellitus (33, 34). Studies in animals and obese patients showed considerably increased levels of IL-6 and TNF-α (36, 37). This increase was likewise observed in our model, with high levels of IL-6 and TNF-alpha among the Zucker fa/fa group. IMMP treatment for 8 weeks decreased IL-6 levels in obese rats. In a previous study of toxic autoimmune hepatitis, IMMP decreased levels of IL-6 in the serum of rats under the same scheme of treatment (16). TNF-α expression has been shown to decrease with the use of synthetic peptides in an in vitro model of oxidative stress (37). However, we did not observe a significant decrease in serum levels among the IMMP treated obese group. In fact, levels of this cytokine increased in the group of slender rats receiving treatment. We must undertake further studies that can help us to identify the physiological impact an increase in this cytokine might have.

IL-1β and IFN-γ are two other cytokines that increase with obesity. IL-1β is a proinflammatory protein involved in metabolic disorders and produced by monocytes and macrophages activated in the form of inactive protein (pro-IL-1β) (38). The expressions of IL-1β and inflammasome components have been found to increase in obese individuals; some of these components are related to the development of metabolic disorders such as insulin resistance (39, 40). IL-1β levels in our Zucker fa/fa rats were high when compared to those of lean animals, whereas IMMP treatment decreased these levels in obese rats. Gamma interferon (IFN-γ) is another cytokine produced by T cells and natural killer (NK) cells the most important function of which is the activation of macrophages in innate immune and adaptive cellular responses. We found a high degree of this cytokine in obese rats and this agrees with other diet-induced or genetically modified models of obesity and diabetes (24, 41). Meanwhile, treatment of these rats with the IMMP resulted in a cytokine decrease. These findings show that the IMMP has an anti-inflammatory effect, since inflammatory proteins decreased in the obese rat group.

Chemokines play an important role in the recruitment of monocytes, neutrophils and lymphocytes for the development of the inflammatory process in obesity. One is monocyte chemoattractant protein-1, better known as MCP-1 or CCL2, which is synthesized by different cell types such as adipocytes and macrophages (42). High levels of this chemokine have been found in adipose tissue and serum in models of obesity and obese or overweight individuals (43, 44), and our result agreed with that. On the other hand, treatment of obese rats with IMMP decreased levels of this chemokine. This effect can be correlated to what has been found in a model of liver damage evaluating IMMP, which decreased the infiltration of inflammatory cells into liver tissue (16). These results and those reported in the literature tell us that one of the mechanisms through which the IMMP decreases tissue macrophages is a reduction in MCP-1 levels. Several studies conducted by our work group support this idea and have shown that IMMP modulates immune system cells in in vitro models, modifying the proliferation of monocytes and lymphocytes (15).

Adipocytes and macrophages in the adipose tissue also secrete anti-inflammatory factors: IL-10 and IL-4 in addition to adiponectin (45). IL-10 is a cytokine with anti-inflammatory properties that is able to inhibit the synthesis of proinflammatory cytokines secreted by T cells and macrophages, whereas IL-4 is an anti-inflammatory cytokine; its presence promotes alternative activation of M2 macrophages and inhibits the classical activation of M1 macrophages, coupled with the secretion of IL-10 and TGF-β, thus reducing inflammation. Our obese rats did not show changes in these cytokines. Since TNF-alpha in this same group also increased slightly, we could infer that the increase in Il-10 and IL-4 was meant to offset the TNF-alpha increase.

In addition to obesity and inflammation, Zucker fa/fa rats present metabolic alterations involving dyslipidemias, light intolerance to glucose and hyperinsulinemia (16). We were able to observe changes in glucose and insulin levels in obese animals, noting hyperglycemia and hyperinsulinemia in the model. Treatment of obese animals did not exert a significant change in biochemical values, but we observed a tendency toward decreased levels of glucose and cholesterol. Zucker fa/fa rats have an overproduction of lipoproteins in the liver, increasing lipids and lipoproteins in plasma. This is reflected in an increase in the levels of triglycerides and blood cholesterol (16). Present result showed that obese rat had high level of triglycerides and cholesterol. Cholesterol levels were unmodified by IMMP treatment, but triglycerides increased in treated animals. This may be due to an increase in the activity of lipoprotein lipase, increasing triglyceride catabolism from adipose tissue, releasing fatty acids into the bloodstream.

The liver plays a central role in the regulation of the glucose and lipid metabolism in the body (46). Triglyceride levels in the liver result from a balance between the ingestion of fatty acids, lipogenesis, and the synthesis of triglycerides and lipoproteins in this organ. The increase of lipids in the generation of steatosis tends to be associated with progressive metabolic deregulation, particularly hepatic insulin alteration and the development of inflammation (47). Non-alcoholic steatohepatitis (NASH) is a common worldwide public health problem and its incidence has increased in the past three decades. This disease is associated with metabolic syndrome factors such as obesity, dyslipidemia, hypertension, and type 2 diabetes (48). Obese animals in this study sustained damage to this organ, with the presence of steatosis and tissue damage. This type of damage can be appreciated in studies with Zucker fa/fa rats and other obesity models induced by diets high in fat and lipopolysaccharides (28, 49). Obese animals treated with IMMP showed a decrease in steatosis and damaged tissue in the liver. This result is consistent with the study of autoimmune hepatic damage, where damage, inflammation and fibrosis in the animals’ liver decreased (16).

In sumary, IMMP exerted an anti-inflammatory effect in an obese Zucker fa/fa rat model, decreasing proinflammatory cytokines in obese animals that received treatment. The decrease of the inflammatory environment could be linked to an improvement of tissue in the damaged liver of obese rats. With our results we can conclude that IMMP could be an effective and safe therapeutic alternative to lessen the inflammatory state associated with metabolic syndrome.
